# *Importance of Aspergillus spp*. isolation in Acute exacerbations of severe COPD: prevalence, factors and follow-up: the FUNGI-COPD study

**DOI:** 10.1186/1465-9921-15-17

**Published:** 2014-02-11

**Authors:** Arturo Huerta, Nestor Soler, Mariano Esperatti, Mónica Guerrero, Rosario Menendez, Alexandra Gimeno, Rafael Zalacaín, Nuria Mir, Jose Maria Aguado, Antoni Torres

**Affiliations:** 1 Department of Pneumology, Institute of Thorax (ICT), Institut d’Investigacions Biomèdiques August Pi i Sunyer (IDIBAPS), Hospital Clinic of Barcelona, University of Barcelona (UB), CIBER de Enfermedades Respiratorias (CIBERES 06/06/0028), Villarroel 170, 08037 Barcelona, Spain; 2 Department of Pneumology, University Hospital La Fe, Valencia, Spain; 3 Department of Pneumology, Hospital Cruces, Byzkaia, Spain; 4 Medical Department Pfizer, Madrid, Spain; 5 Unit of Infectious Diseases, Hospital 12 de Octubre, University Complutense, Madrid, Spain

## Abstract

**Background:**

Acute exacerbations of COPD (AECOPD) are often associated with infectious agents, some of which may be non-usual, including *Aspergillus* spp. However, the importance of *Aspergillus* spp. in the clinical management of AECOPD still remains unclear.

**Objectives:**

The aims of the study were to analyze the prevalence and risk factors associated with *Aspergillus* spp*.* isolation in AECOPD, and to investigate the associated clinical outcomes during a 1-year follow-up period.

**Methods:**

Patients presenting with an AECOPD requiring hospitalization were prospectively included from four hospitals across Spain. Clinical, radiological and microbiological data were collected at admission and during the follow-up period (1, 6 and 12 months after discharge), and re-admissions and mortality data collected during the follow-up.

**Results:**

A total of 240 patients with severe AECOPD were included. Valid sputum samples were obtained in 144 (58%) patients, and in this group, the prevalence of *Aspergillus* spp*.* isolation was 16.6% on admission and 14.1% at one-year follow-up. Multivariate logistic-regression showed that AECOPD in the previous year (OR 12.35; 95% CI, 1.9-29.1; p < 0.001), concurrent isolation of pathogenic bacteria (OR 3.64; 95% CI 1.65-9.45, p = 0.001) and concomitant isolation of *Pseudomonas aeruginosa* (OR 2.80; 95% IC, 1.81-11.42; p = 0.001) were the main risk factors for *Aspergillus* spp. isolation.

**Conclusions:**

The main risk factors for *Aspergillus* spp. isolation were AECOPD in the previous year and concomitant isolation of *Pseudomonas aeruginosa*. However, although *Aspergillus* spp. is often isolated in sputum samples from patients with AECOPD, the pathogenic and clinical significance remains unclear.

## Introduction

Exacerbations of chronic obstructive pulmonary disease (COPD) are frequent events in the natural history of the disease, increasing mortality especially when patients require hospitalization [1,2]. Acute exacerbations of COPD (AECOPD) are characterized clinically by worsening of dyspnoea, increased sputum production and/or changes in sputum purulence [3]. There is evidence suggesting that some infectious agents, typically respiratory viruses and bacteria, increase bronchial and systemic inflammation, which is commonly seen in AECOPD [4,5].

Overall, these microbial agents account for the etiology of 75% of AECOPD, particularly in Anthonisen Type I exacerbations [6]. However, the role of non-usual microorganisms such as *Aspergillus* spp. has not been well established. Previous published data investigating *Aspergillus* spp. in COPD are retrospective [7] or were conducted in a small series of patients [8]. However, *Aspergillus* spp. may be responsible for important clinical events from saprophytic colonization of the airways to rapidly invasive and life-threatening disseminated diseases, depending on the host immune status and the presence of underlying lung disease [9]. Patients with severe COPD who often receive broad-spectrum antibiotics and corticosteroids are now acknowledged to be one of the main risk groups for pulmonary aspergillosis [10,11].

As little is known about the risk of pulmonary aspergillosis in severe COPD patients, some retrospective studies have analyzed the incidence of *Aspergillus fumigatus* isolation from lower respiratory tract samples in non-immunocompromised patients and shown that COPD patients are an important group which are affected by either colonization or proven aspergillosis [12].

In one of the largest studies investigating the prevalence of *Aspergillus* spp. from *Aspergillus* respiratory samples in a cohort of critically ill patients, positive cultures *Aspergillus* occurred in 36 out of a total of 1756 patients, and *Aspergillus* treatment with steroids (odds ratio (OR) = 4.5) and chronic obstructive pulmonary disease (OR = 2.9) were significantly associated with *Aspergillus* spp. isolation in their multivariate analysis [13]. However, it remains unclear why some COPD patients are colonized by *Aspergillus* spp. whereas others develop invasive pulmonary disease. Colonization may correspond to a temporary passage of *Aspergillus* spp. in the airways, long-term benign carriage, or a sign of preceding invasive disease [14-17]. However, the absence of prospective studies designed to determine the prevalence of Aspergillus spp. airway colonization *Aspergillus*in patients with AECOPD means that it has not been possible to define the influence of this organism as a causal agent of exacerbations.

Thus, the aim of this study was to analyze the prevalence, risk factors and clinical evolution associated with the respiratory isolation of *Aspergillus* spp. in a cohort of COPD patients requiring admission to the hospital with an AECOPD.

## Methods

### Study data and design

Data was prospectively collected from patients hospitalized due to a severe COPD exacerbation in four tertiary university teaching hospitals in Spain between January 2008 and December 2009. Ethics Approval Committee (CEIC 2008/4325) and all patients provided written informed consent.

### Definitions

A complete definition of COPD, severe COPD exacerbation and pulmonary *Aspergillus* spp. infection.

### Study protocol

Only COPD patients with GOLD confirmed by spirometry were included. Patients were evaluated for inclusion in the study within the first 24 hours after admission to the Emergency Department. Diagnosis of COPD exacerbation, decision to hospitalize, time of discharge and choice of pharmacological therapy were taken by the physician in charge. Patients with active tuberculosis, asthma, immunosuppression (innate or acquired) or any other clinical respiratory diseases were excluded. All patients discharged after an AECOPD were scheduled for follow-up visits at one, six and 12 months. Each patient was only included once in the analysis, despite the possibility of more than one re-admission during the follow-up period.

### Data collection

Demographic variables, presence of any comorbid conditions (heart, renal, neurologic and liver disease, diabetes or cancer), smoking status, perceived dyspnoea and use of pharmacotherapy (including COPD baseline treatment, antibiotics, and anti-pneumococcal and/or flu vaccination) were recorded on admission to hospital. Symptoms/signs of the AECOPD together with physiological and laboratory data were collected at onset. Other variables such as length of stay (LOS), frequency of patients in whom admission to the intensive care unit (ICU) was needed, or requirement of non-invasive mechanical ventilation (NIMV) were also recorded.

The number of AECOPD events in the year preceding hospitalization was assessed based on recorded data and a search of the database of all hospital contacts. Only exacerbations requiring emergency room visits or admissions were included.

### Microbiological analysis

Spontaneous sputum samples were obtained on admission and in each follow-up visit where possible. Sputum was examined for leukocytes and epithelial cells by gram stain, and samples with a Murray-Washington classification criteria IV (10-25 epithelial cells and >25 leukocytes per field) or V (≤10 epithelial cells and >25 leukocytes per field) were considered representative of distal airways and subsequently processed for culture. Bacterial growth was assessed at 48 hours and fungal growth after 72 hours. The airway bacteria cultured were classified into potential pathogenic microorganisms (PPMs) and non-PPMs, as previously described [5]. Samples were processed by the reference clinical microbiology laboratories of the participating hospitals using standard procedures, including Sabouraud agar culture, for the isolation of fungal species. Cultures were incubated at 32°C-37°C for at least 7 days and the number of visible colonies recorded. After seven days, filamentous colonies were examined and *Aspergillus* spp. identified based on macroscopic and microscopic methods.

### Statistical analysis

Data were analysed using SPSS version 17.0 for Windows (SPSS Inc., Chicago, IL, USA). Categorical variables are presented as absolute numbers and relative frequencies, while continuous variables are presented as the mean, standard deviation (SD) in parametric data, or median with the interquartile (IQR) range in non-parametric data. Categorical variables were compared using the χ^2^ test or Fisher’s exact test, as appropriate. For the comparison of continuous variables, the *t* test was used if normality was demonstrated; otherwise, the non-parametric Mann-Whitney U test was performed.

In order to determine the risk factors associated with the presence of *Aspergillus* spp. in the airways, we used both univariate and multivariate logistic regression model, where the dependent variable was the isolation of *Aspergillus* spp. in patients at admission, and independent variables were those associated with demographics and clinical factors. The association between follow-up visits was analyzed using the Wilcoxon test for quantitative variables and McNemar test for dichotomous discrete variables. We checked the assumptions of normality necessary to use parametric tests. All tests were two-tailed and significance was set at 5%. Bonferrioni correction was used when needed.

## Results

**Figure 1:**
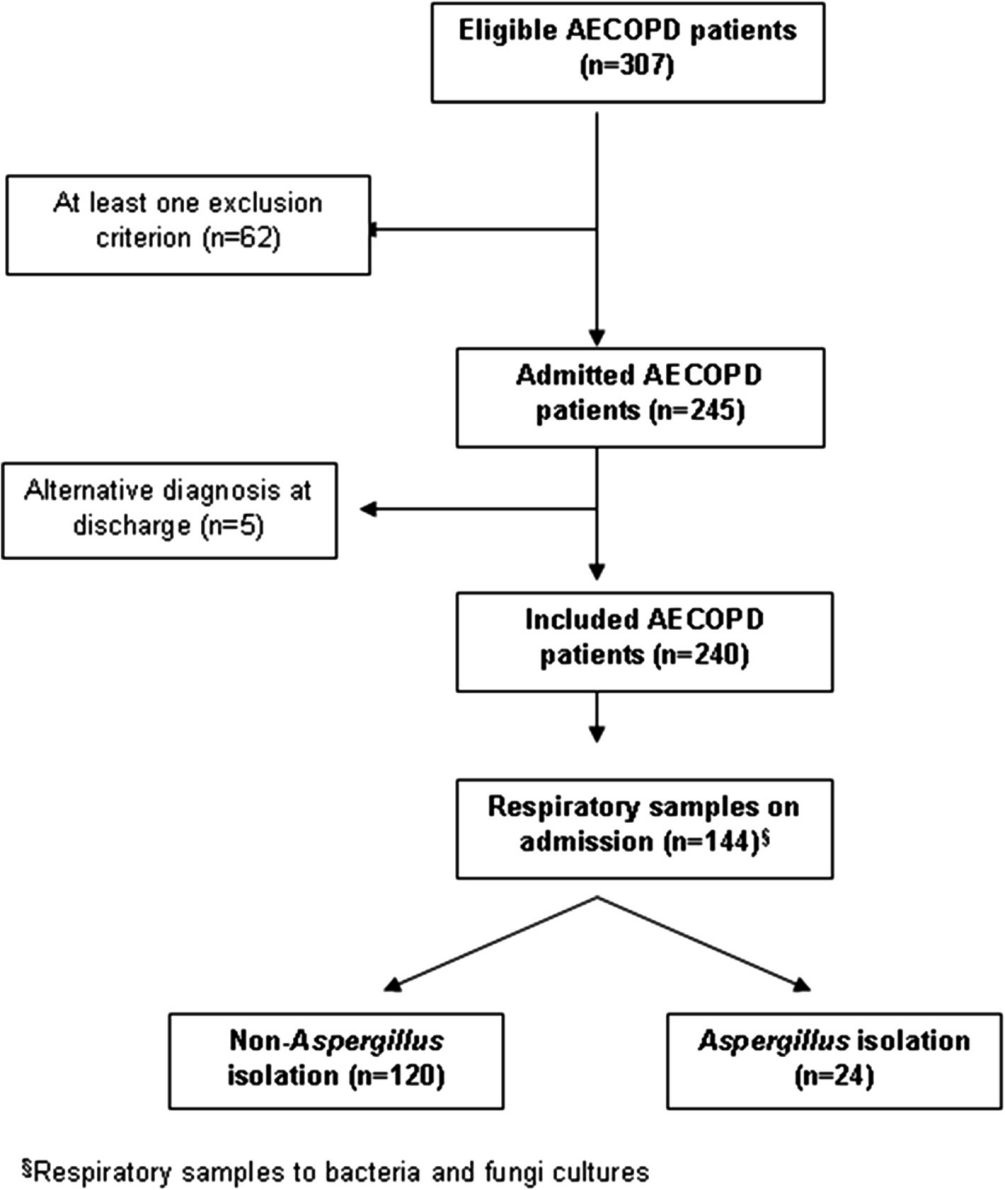
Flow diagram of patient inclusion.

A total of 307 possible eligible patients presenting to the emergency department with a severe exacerbation of COPD were evaluated. Of these, 62 patients presented with one or more exclusion criteria (Figure 1). In the remaining 245 cases, an alternative diagnose was recorded at discharge in 5 patients (2%) and they were excluded from the analysis. Therefore, the study population included for analysis comprised of 240 patients.

### Baseline and clinical characteristics

The cohort recruited consisted predominantly of men (217 [90%]; mean ± SD age 74.4 ± 8.8 years) and current smokers (172 [72.6%]). The patients had evidence of severe airway obstruction (mean FEV_1_ of 44.0 ± 14.6% predicted). Previous history of COPD exacerbations was very common, with 64.2% of patients having had at least one severe exacerbation in the year preceding the index hospitalization (mean 2.88 ± 2.27, maximum 6 exacerbations), and among this group, 39% were re-admitted to the hospital.

Of all the patients included, 170 (70.8%) had previously received pharmacological treatment for their COPD with a fixed combination of long acting β_2-_agonists (LABA) and inhaled corticosteroids (ICS): formoterol plus budesonide in 52 patients (30.6%) and fluticasone plus salmeterol in 118 patients (69.4%). 174 (72.5%) patients had received the influenza vaccine and 101 (42.1%) had received the pneumococcal vaccine.

Analysis of bacterial colonization in the previous year showed that 85 patients (35.4%) had microbiological evidence of bacterial colonization in the year prior to inclusion. The clinical characteristics of the overall population included patients by *Aspergillus* spp. isolation is shown in Table 1. There was no significant difference in clinical characteristics between the two groups (*Aspergillus* spp. isolation *versus* no *Aspergillus* spp. isolation). The clinical, physiological and laboratory features of the AECOPD on admission and the subsequent outcomes of the patients are shown in Table 2.

**Table 1:** **Characteristics of the study population on admission based on *Aspergillus spp. *** **isolation**

	**All patients (n = 240)**	**No *Aspergillus *** **spp. isolation (n = 120)**^**#**^	***Aspergillus *** **spp. isolation (n = 24)**^**#**^	**p-value†**
Age, years	74.4 ± 8.8	74.2 ± 10.1	73.1 ± 8.2	0.569
Gender, males	217 (90.4)	103 (85.8)	23 (95.8)	0.246
Past-smoker	175 (72.6)	87(72.5)	18 (75.0)	0.608
Current smoker	65 (27.4)	33 (27.5)	6 (25.0)	0.779
FEV_1_% predicted	2.6 (1-3)	47.3 ± 12.9	45.4 ± 14.7	0.196
FVC% predicted	44.0 ± 14.6	71.9 ± 14.4	68.8 ± 16.4	0.094
*Charlson index*	69.6 ± 19.6	2.1 (1.3-3.4)	2.7 (1.6-3.9)	0.060
GOLD stage II	12 (5,8)	8 (6.6)	**-**	**-**
GOLD stage III	92 (38,3)	46 (38.3)	8 (33.3)	0.436
GOLD stage IV	136 (56,1)	66 (55.5)	16 (66.6)	0.062
COPD exacerbation in the previous year				
≤ 2 exacerbations	82 (53.2)	65 (63.7)	7 (38.8)	0.150
>2 exacerbations	72 (46.7)	37 (36.3)	11 (61.1)	0.050
Admission for COPD in the previous year	2.6 ± 1.9	2.1 ± 0.8	2.4 ± 1.2	0.079
Long Term Oxygen Therapy	72 (30.0)	36 (29.1)	9 (37.5)	0.067
Cardiovascular disease^1^	131 (54.6)	61 (50.8)	12 (50.0)	0.475
Arterial hypertension^2^	112 (46.7)	52 (43.3)	19 (79.2)	0.002
Diabetes mellitus	65 (27.1)	31 (25.8)	9 (37.5)	0.206
Chronic renal disease	34 (14.2)	15 (12.5)	6 (25.0)	0.052
Inhaled corticosteroids	170 (70.8)	85 (70.8)	18 (75.0)	0.763
<500 mcg daily	106 (62.3)	54 (43.5)	12 (66.6)	0.096
>500 mcg daily	64 (37.6)	31 (36.5)	6 (33.3)	0.267
Oral corticosteroids	80 (33.3)	39 (32.5)	9 (37.5)	0.284

Data are presented as means ± SD, medians (Interquartile range) or absolute numbers (%), as appropriate. ^1^Patients with cardiovascular disease other than arterial hypertension; ^2^Patients with arterial hypertension without any other cardiovascular condition. ^#^Data calculated from the total number of respiratory samples. † p-value refers only to the comparison between non-aspergillus and *Aspergillus* isolated populations.

**Table 2:** Clinical characteristics of AECOPD at admission and outcomes

	**All patients**	**No *Aspergillus *** **spp. isolation**	***Aspergillus *** **spp.isolation**	**p-value †**
	**(n = 240)**	**(n = 120)**^**#**^	**(n = 24)**^**#**^	
Heart rate, beats/min	96.8 ± 16.1	94.7 ± 14.8	95.6 ± 13.4	0.624
Respiratory rate, breath/min	23.3 ± 8.9	22.4 ± 9.7	24.7 ± 7.6	0.286
Body temperature, °C	36.7 ± 0.9	36.4 ± 0.8	36.8 ± 0.9	0.574
*Type of exacerbation*^£^				
Type I	110 (45.8)	56 (46.6)	10 (41.6)	0.340
Type II	59 (24.3)	30 (25.0)	5 (20.8)	0.127
Type III	81 (33.4)	34 (28.3)	9 (37.5)	0.193
*Laboratory findings*				
Leucocytes, x 10^9^/L	11.8 ± 6.3	11.2 ± 5.9	12.0 ± 4.3	0.391
C-reactive protein, mg/dL	3.2 (1.2-12.3)	2.9 (1.2-13.5)	3.4 (1.6-9-4)	0.192
pH	7.376 ± 0.064	7.401 ± 0.035	7.363 ± 0.078	0.286
PaO_2_, mmHg	63.1 ± 22.7	64.6 ± 15.7	61.5 ± 17.9	0.141
PaCO_2_, mmHg	47.8 ± 13.3	45.5 ± 12.8	49.4 ± 17.2	0.096
MV^§^ requirements on admission	9 (3.7)	8 (6.6)	1 (4.2)	0.297
Length of stay, days	9.6 ± 6.4	7.5 ± 5.0	11.8 ± 9.2	0.025
*30-days follow-up*				
Exacerbations, n	58 (24.1)	29 (24.1)	6 (25.0)	0.540
Hospital re-admissions	34 (14.1)	17 (14.2)	3 (12.5)	0.345
Death by any cause	11 (4.6)	6 (5.0)	1 (4.2)	0.478
*12-month follow-up*				
Exacerbations	131 (54.5)	72 (60.0)	12 (54.4)	0.299
Hospital re-admissions	93 (38.7)	52 (43.3)	7 (31.8)	0.087
ICU admissions	14 (5.8)	8 (6.6)	2 (9.0)	0.065
Death by any cause	27 (11.3)	13 (10.8)	3 (12.5)	0.373

Data are presented as means ± SD, medians (1^st^ quartile; 3^rd^ quartile) or numbers (%) as appropriate. ^#^Data calculated from the number of valid samples. ^£^Anthonisen exacerbation criteria [4]; ^*£*^*MV*, Mechanical ventilation. † p-value refers only to the comparison between non-aspergillus and *Aspergillus* isolated populations.

### Microbiological findings and prevalence of Aspergillus isolation

Sputum of sufficient quality for bacterial and fungal culture was obtained from 144 patients on admission. During the initial admission and the subsequent follow-up visits, a total of 198 sputum samples were collected. Non-PPMs were isolated in 112 (57%), whilst PPMs were found in 86 (43%). *Pseudomonas aeruginosa* was the most frequently isolated PPM (27%). The full distribution of PPMs is shown in Table 3. 28 (14%) out of the 198 sputum samples had *Aspergillus* spp. isolated from 24 COPD patients. Only four patients, had *Aspergillus* spp. isolated in two different samples during the follow-up period. *Aspergillus fumigatus* was isolated in 25 cases, *Aspergillus niger* in two, and *Aspergillus flavus* in one.

**Table 3:** **Cumulative identification and incidence of potential pathogenic microorganisms (PPM) cultured from COPD patients**^**£**^

	**No *Aspergillus *** **spp. isolation**	***Aspergillus *** **spp. isolation**	**Total**	**p-value**
**Sputum samples**	170	28	198	
**Samples with PPM**^**§ **^**cultures**	66 (38.8)	20 (71.4)	86 (43)	0.024
*Haemophilus influenzae*	13 (7.6)	5 (17.8)	18 (9.1)	0.246
*Streptococcus pneumoniae*	11 (6.4)	2 (7,1)	13 (6.6)	0.626
*Staphylococcus aureus*	9 (5.3)	3 (10.7)	12 (6.1)	0.372
*Moraxella catarrhalis*	9 (5.3)	1 (3.6)	9 (5.1)	0.564
*Enterobacter spp.*	3 (1.8)	1 (3.6)	4 (2.0)	0.632
*Klebsiella pneumoniae*	2 (1.2)	-	2 (1.1)	-
*Escherichia coli*	4 (2.3)	-	4 (2.0)	-
*Serratia marcescens*	2 (1.2)	-	2 (1.1)	-
*Pseudomonas aeruginosa*	14 (8.2)	9 (32.1)	23 (11.6)	0.042
*Stenotrophomonas maltophilia*	2 (1.1)	-	2 (1.1)	-
*Aspergillus fumigatus*	-	25 (89.3)	25 (12.6)	-
*Aspergillus* spp.*	-	3 (10.7)	3 (1.5)	-
**Samples with non-PPM**^**# **^**cultures**	104 (61.2)	8 (28.6)	112 (56.6)	0.063
*Haemophilus parainfluenzae*	14 (8.2)	2 (7.1)	16 (8.1)	0.674
*Streptococcus viridans*	41 (24.1)	2 (7.1)	43 (21.7)	0.135
*Staphylococcus epidermidis*	14 (8.2)	-	14 (7.1)	-
*Corynebacterium* spp*.*	8 (4.7)	-	8 (4.0)	-
*Neisseria* spp*.*	6 (3.5)	-	6 (3.0)	-
Mixed upper airways flora	21 (12.3)	4 (14.3)	25 (12.6)	0.78

^**£**^All comparisons have been calculated in valid sputum samples obtained in each group. *Aspergillus spp*.: Aspergillus niger (2), Aspergillus flavus (1);*^*§*^*PPM*, Pathogenic microorganisms; ^*#*^*non-PPM*, Non-pathogenic microorganisms.

The prevalence of *Aspergillus* spp. isolation in this cohort of COPD patients was 16.6% on admission and 14.1% at the end of the follow-up period.

Due to clinical concern that doubtful pulmonary infiltrates seen on chest X-ray at admission may represent possible invasive pulmonary aspergillosis (IPA), according to Bulpas’s criteria (16), a high resolution computed thoracic (HRCT) scan was performed in 18 of the 24 patients (75%) with *Aspergillus* isolation. No features of IPA were observed in any patient. Nevertheless, in five patients, their treating physicians decided to start antifungal treatment due to previous exposure to oral corticosteroids (voriconazole in three cases and itraconazole in two).

### Risk factors for Aspergillus isolation

Previous bacterial colonization, the number of previous COPD exacerbations that required hospital visits (emergency room visit or admission) and the presence of arterial hypertension were significantly more frequent in patients with *Aspergillus* spp. isolation. Patients with *Aspergillus* spp. isolation in their sputum samples, had experienced more than two previous episodes of AECOPD compared to those patients without *Aspergillus* spp. isolation (61 *versus* 11, p = 0.05).

Analysis of the microbiological data in the overall population prior to admission, showed a significant association between previous bacterial colonization and the isolation of *Aspergillus spp*. (42 *versus* 11, p = 0.002). Similarly, there was a significant association between the presence of PPMs in sputum samples collected during the study period and concurrent isolation of *Aspergillus* spp compared to those patients without PPMs but with *Aspergillus* spp. isolation (66 *versus* 20, p = 0.02). This difference was also significant in cases with previous isolation of *Pseudomonas aeruginosa* (14 *versus* 9, p = 0.04).

Table 4 summarizes the results of the multivariate analysis of risk factors potentially associated with *Aspergillus* spp. isolation. Significant variables associated independently with *Aspergillus* spp. isolation were arterial hypertension (OR 4.72; 95% confidential interval (CI), 1.56-14.29; p < 0.001), COPD exacerbation that required hospitalization in the last year (OR 12.35; 95% CI, 1.87-29.14; p < 0.001), concurrent PPMs isolation (OR 3.64; 95% CI, 1.65-9.45; p = 0.001), and concomitant isolation on the same sample of *Pseudomonas aeruginosa* (OR 2.80; 95% CI, 1.81-11.42; p = 0.001).

**Table 4:** **Significant univariate and multivariate logistic regression analysis for the prediction of *Aspergillus *** **spp. risk factors**

	**Univariate analysis**			**Multivariate analysis**		
	**OR**	**95%CI**	**p-value**	**OR**	**95%CI**	**p-value**
Arterial hypertension	6.48	1.17-35.84	0.032	4.72	1.56-14.39	0.001
Cardio vascular disease	9.97	1.30-76.47	0.027	**-**	**-**	**-**
Diabetes mellitus	3.52	1.77-15.94	0.041	-	-	-
Chronic renal disease	2.92	2.23-15.35	0.070	**-**	**-**	**-**
COPD exacerbation in the previous year ^§^	2.60	1.16-6.11	0.050	12.35	1.87-29.14	<0.001
Previous bacterial colonization	3.50	1.47-8.37	0.002	-	-	-
Concurrent PPM isolation ^£^	2.92	1.42-6.84	0.024	3.64	1.65-9.45	0.001
Concurrent *P. aeruginosa* isolation	2.10	1.21-5.68	0.042	2.80	1.81-11.42	0.001

^§^COPD exacerbation that required hospital management (emergency room visit or admission).

^£^*PPM*, Pathogenic microorganisms.

### Short-term and long-term clinical outcomes

The short- and long-term clinical outcomes are shown in Table 2. Patients with *Aspergillus* spp. isolation had significantly higher LOS compared to those patients without *Aspergillus* spp. isolation (7.5 ± 5.0 days *Aspergillus versus* 11.8 ± 9.2 days *Aspergillus*, p = 0.02). No differences were found in the rate of exacerbations within the first month (24% versus 25%; p = 0.54) or during the follow-up (60% versus 54%; p = 0.34).

The overall mortality rate at 12 months was 11.3% (27 patients); 13 (10.8%) in the non-*Aspergillus* spp. group and three (12.5%) in the *Aspergillus* spp. group (p = 0.373).

## Discussion

This is the first study to date to prospectively determine the prevalence of airway *Aspergillus* spp. and examine the associated risk factors for isolation, in a cohort of severe COPD patients requiring hospitalization for an AECOPD. We have shown that the prevalence of *Aspergillus* spp. isolation in this cohort was 16.6% on admission and 14.4% at the end of one-year follow-up. The independent risk factors associated with *Aspergillus* spp. colonization were an AECOPD in the previous year, and the concurrent isolation of PPMs, most frequently *Pseudomonas aeruginosa*. Notably, *Aspergillus* spp. isolation was not associated with more severe exacerbations or worse clinical outcomes after a one-year follow up.

We found 28 *Aspergillus* spp. isolates using a standard method for mycological investigations in spontaneous sputum samples. The prevalence rate of 14.4% in our COPD cohort after a one-year follow-up was surprisingly high. In fact, *Aspergillus* spp. was the most frequent microorganisms isolated, together with *Pseudomonas aeruginosa*. Importantly, we found significant differences in the bacterial pathogens isolated in the patients with and without *Aspergillus* spp. isolation, independent of COPD severity, suggesting an increase in the rate of co-infection in COPD patients who present with *Aspergillus* spp. isolation.

The prevalence of *Aspergillus* spp. isolation may have been higher if we had used bronchoscopic techniques and specific culture media. However, in real-life settings, clinicians often only have access to sputum samples. In a recent study, Phasley *et al*. [18] reported that the isolation of *Aspergillus fumigatus* in sputum culture was significantly higher using a research approach compared to the standard method for mycological investigations. Previous studies, which have not focused solely on *Aspergillus* spp., have found different prevalence rates of fungi isolation in respiratory samples from patients with cystic fibrosis, COPD and asthma [19-21]. Recently, a large, retrospective study conducted by Guinea *et al.*[7], analyzed the incidence of *Aspergillus fumigatus* isolation from lower respiratory tract samples in patients admitted for AECOPD in a tertiary hospital The authors found 239 isolations of *Aspergillus* spp. (16.3 per 1000 admissions), but only 53 (22%) patients had probable IPA. However, unlike our prospective study, the fungal isolations were detected retrospectively by the microbiology laboratory.

There is no doubt that COPD patients are a population at risk for *Aspergillus* spp. colonization [12,14]. In a previous study of critically ill patients, *Aspergillus* spp. isolation from respiratory secretions was significantly associated with both an underlying diagnosis of COPD and treatment with corticosteroids [13]. These findings have been confirmed by other authors, and have strengthened the relationship between pulmonary infection with *Aspergillus* spp. and the use of intravenous corticosteroids in COPD patients admitted to the ICU for severe exacerbation [16]. In contrast, a study conducted by Afessa *et al.*[22] reported no isolation of *Aspergillus* spp. in the respiratory specimens from 250 COPD patients admitted to the ICU because of acute respiratory failure, although no report on corticosteroid therapy was performed.

The concurrent isolation of other pathogens, especially *Pseudomonas aeruginosa* was also associated with a higher risk of *Aspergillus* spp. isolation. Although the role of bacterial infection in COPD exacerbations remains controversial, *Pseudomonas aeruginosa* is usually isolated in patients with advanced COPD stages [23-25]. In addition, the incidence of *Pseudomonas aeruginosa* in sputum samples from patients hospitalized with COPD exacerbations is high, and its isolation could also be a marker of poor prognosis independently of other predictors [26].

Patients with previous severe exacerbations are more likely to receive a higher number of antibiotic therapy courses, and this could be the key factor in promoting further *Aspergillus* spp. isolation. In fact, in a recent publication, it was found that almost 73% of COPD patients in Spain presenting with a Type I Anthonisen exacerbation received antibiotics [27]. Prospective studies focusing on this specific population are much needed to determine whether *Aspergillus* spp. isolation is the cause or the consequence of more infectious exacerbations with concurrent isolation of *Pseudomonas aeruginosa*. Paradoxically to the data of Bafhadel *et al.*[28]*,* we found no association between the use of either inhaled (ICS) or oral (OCS) corticosteroids and an increased rate of *Aspergillus* spp.

Colonization of the distal airways by filamentous fungi, such as *Aspergillus spp.,* can occur during the course of COPD, although the clinical relevance is unclear. To date, it is unknown whether colonization by *Aspergillus* spp. contributes to the increased frequency and severity of exacerbations, or is a marker of more severe disease. In our study, except for a longer LOS, we found no significant differences in any of the other clinical outcomes when comparing patients with and without *Aspergillus* spp. isolation. Therefore, antifungal treatment may not be beneficial in these patients. Only five of our patients were treated with antifungals despite the fact that none of the CT scans performed showed radiological signs of pulmonary aspergillosis but only doubtful infiltrates on chest x-ray at admission. Importantly, we followed up patients for one year, and there was no significant difference in ICU-admissions and hospital readmissions between the non-*Aspergillus* and *Aspergillus* spp. isolation groups, again suggesting that *Aspergillus* spp. detection does not worsen clinical outcomes.

Several limitations of our study require attention. Firstly, we only used sputum samples for culture, and it can be argued that the value of these specimens is poor in identifying the etiologic agent of the exacerbation. Obtaining sputum samples is a real-life cost-effective tool, but may have limited value in distinguishing transitional *Aspergillus* spp. isolation from chronic colonization. However, in a previous bronchoscopic validation study published by our group, we found a high concordance between fungal pathogens in samples retrieved via protected specimen brush and sputum (κ = 0.85, p < 0.002) [29]. Although there are new microbiological techniques for the rapid detection of *Aspergillus* spp. that provide results in less than 4 hours (compared to 3 or more days using conventional culture) [30], until they become widely available, conventional sputum culture remains a good diagnostic tool for *Aspergillus* spp. identification. Secondly, this study was performed in patients admitted with a severe AECOPD in Spain, and bacteria and fungi flora may differ in different countries. Thirdly, we did not routinely perform CT scans on admission to all patients with *Aspergillus* spp. isolation. However, none of the 18 patients who were evaluated by a CT scan showed any radiological signs of invasive or chronic pulmonary aspergillosis. Finally, we did not investigate plasma galactomannan antigen, and are unaware of the value of this pathogenic marker in severe COPD patients with *Aspergillus* spp. isolation. In a recent study of critically ill patients, bronchoalveolar lavage (BALF) galactomannan had a higher sensitivity in the early diagnosis of IPA but its value still remains unclear since the collection and processing of BALF samples for *Aspergillus* spp. has not yet been standardized [31]. In another study, the cut-off of value for Platelia galactomannan of 1 ng/ml was the best for diagnosing invasive aspergillosis, with both high sensitivity and specificity [32].

In summary, we found a high prevalence of *Aspergillus* spp. isolation in a cohort of COPD patients with severe AECOPD requiring hospitalization. Except for a longer LOS, we found no differences in the short-term or long-term outcomes. We detected clinical and microbiological risk factors for *Aspergillus* isolation. However, the infective role of these isolates seems to be minimal. Our findings may serve to propose future more comprehensive studies, with a biological basis that includes pulmonary and systemic markers of the immune and inflammatory response, in order to determine the role of this fungus in COPD exacerbations.

### Ethics approval

Ethics approval was provided by the Research Committee from the Hospital Clinic de Barcelona (CEIC 2008/4325).

## Competing interests

This work in original and all authors meet the criteria for authorship, including acceptance of responsibility for the scientific content of the manuscript. NM is actually employed by Pfizer Pharmaceuticals Spain but did not influence the interpretation of the data analysis.

## Authors’ contributions

Study concept and design: NS, AT. Coordination of the acquisition of the data: AH, ME, AM, MG, RZ. Data analysis: NS, AH, NM. Data interpretation: NS, AT, NM, JMA. Writing the article: AH, NS, AT. Critical revision of the manuscript: AH, AT, RM, NS. All authors read and approved the final manuscript.

## Support statement

This study was funded by Institut d’Investigacions Biomèdiques Agusti Pi i Sunyer (IDIBAPS), CIBER de Enfermedades Respiratorias (CIBERES CB06/06/0028) an Initiative of The Institute de Salud Carlos III (ISCIII) and also sponsored by Pfizer clinical protocol A1501086.
